# Enhancing Channel Contention Efficiency in IEEE 802.15.4 Wireless Networks

**DOI:** 10.3390/s22041600

**Published:** 2022-02-18

**Authors:** Yi-Hua Zhu, Luming Jia, Yufan Zhang

**Affiliations:** The School of Computer Science and Technology, Zhejiang University of Technology, Hangzhou 310023, China; jialuming953@163.com (L.J.); 1112012016@zjut.edu.cn (Y.Z.)

**Keywords:** IEEE 802.15.4 standard, CSMA-CA, IoT, channel access efficiency

## Abstract

Numerous Internet of Things (IoT) devices adopt the IEEE 802.15.4 standard, which targets low data rate wireless networks. With the explosive growth in the use of IoT devices, it is essential to design effective and efficient channel access schemes for the 802.15.4 networks. In order to improve channel contention efficiency (CCE), which is defined as the number of times of successfully gaining the channel per unit of backoff time whereby throughput is improved, the scheme of enhancing channel contention efficiency (ECCE) has been proposed to jointly optimize the three key parameters of *macMinBe*, *macMaxBe* and *macMaxCsmaBackoffs* in the carrier sense multiple access with collision avoidance (CSMA-CA) mechanism in the 802.15.4 standard. A novel Markov chain was developed to model the CSMA-CA mechanism, which yielded the expected number of failures in gaining the channel, the expected number of backoff periods and the expected number of backoffs when a node intended to transmit a packet. These statistics resulted in CCE. An optimization problem that maximized the CCE with respect to the above-mentioned three key parameters was formulated. The solution to the optimization problem led to the optimal parameter values, which were applied in the ECCE scheme. The simulation results show that the proposed ECCE scheme outperformed the CSMA-CA mechanism in terms of CCE, delay and throughput.

## 1. Introduction

The IEEE 802.15.4 standard, initiated in 2006, defines the physical (PHY) layer and medium access control (MAC) sublayer specifications for low data rate wireless connectivity for nodes with no battery or with very limited battery consumption [1]. The standard was updated in 2011 [2], 2015 [3] and 2020 [4], i.e., the last version of the IEEE 802.15.4 standard was released in 2020.

Contemporary Wireless Sensor Networks (WSNs) are main components of the Internet of Things (IoT) and as such, adopt the IEEE 802.15.4 standard in both MAC and PHY layers [5]. Nowadays, with the explosive growth of the number of devices injected into the IoT, IoT devices operating with the 802.15.4 standard face increasingly serious competition in gaining the channel. Therefore, it is important to enhance the channel contention efficiency for 802.15.4 wireless networks.

To date, there have been many studies on channel contention in 802.15.4-based networks in the literature. These works take channel access time, clear channel assessment (CCA) results, the state of busy or idle channels, packet collision, transmission failure rate and other factors into account in order to improve throughput, reduce delay, enhance energy efficiency, ensure high reliability or provide fair channel access.

In fact, in the IEEE 802.15.4 standard, the carrier sense multiple access with collision avoidance (CSMA-CA) channel access mechanism was introduced for the network nodes to contend for the channel. The CSMA-CA mechanism is classified into two categories: slotted CSMA-CA and unslotted CSMA-CA. This paper focuses on the former. There are three key parameters in the slotted CSMA-CA mechanism that considerably impact channel contention efficiency, namely *macMinBe*, *macMaxBe* and *macMaxCsmaBackoffs*. Unfortunately, the IEEE 802.15.4 standard only defines the ranges of these parameters, leaving their optimal values unresolved. As a result, the nodes in IEEE 802.15.4 standard-based networks are faced with the dilemma of how to set appropriate values for these parameters. Hence, it is desirable to design a scheme that enables a node to set the optimal values of these parameters so that channel contention efficiency is improved. This was the motivation for this paper. To the best of our knowledge, this is the first study that jointly optimizes the three key parameters to improve channel contention efficiency (CCE).

The main contributions of the paper are as follows:

- We propose the scheme of enhancing channel contention efficiency (ECCE), aiming to improve throughput and reduce delay through maximizing the CCE, which is defined as the number of successful gaining the channels per unit of backoff period;
- We developed a novel Markov chain to model the CSMA-CA, which yielded the following statistics: the expected number of failures in gaining the channel per packet, the expected number of backoff periods and the expected number of backoffs that a node experiences for transmitting a packet. These statistics were used to calculate the CCE. In addition, we formulated an optimization problem that maximized the CCE with respect to the aforementioned three key parameters in the CSMA-CA mechanism. The solution to the optimization problem leads to the optimal values of the parameters, which are applied in the proposed ECCE scheme;
- The simulation results show that the proposed scheme outperformed the CSMA-CA mechanism in terms of CCE, throughput and delay.

The remainder of this paper is organized by follows. The related works are surveyed in Section 2. In Section 3, we introduce the background and present the definition of the problem. In Section 4, we propose the ECCE scheme with its Markov model and formulate the optimization problem. In Section 5, we evaluate the performance of the proposed scheme via simulation. We conclude the paper in Section 6.

## 2. Related Work

Channel access that is based on the CSMA-CA has been intensively investigated within the research community. Z. Tao et al. [6] used a Markov chain to model the CSMA-CA mechanism in IEEE 802.15.4 networks and pointed out that a slight modification to the protocol can considerably improve throughput and delay. J. Abegunde et al. [7] presented a game theory-based solution to 802.15.4 channel contention, which reduces energy consumption and increases fairness. Z. Zhang et al. [8] applied a priority mechanism and virtual carrier sensing to the traditional CSMA-CA algorithm to improve throughput and reduce energy consumption. F. Shu et al. [9] analyzed the throughput of the energy conserving CSMA-CA under saturated and periodic traffic conditions. Y. Zhang et al. [10] studied the idle period after the last collision and proposed a mechanism to save power and improve throughput. C. Wang et al. [11] showed that the optimization of channel access time can reduce energy consumption. In fact, channel access time, or the backoff periods in the CSMA-CA mechanism, also affects throughput, delay, etc. Some parameters in the CSMA-CA mechanism that affect channel access time have been investigated. S. Brienza et al. [12] optimized them in terms of reliability and energy efficiency. J. Y. Ha et al. [13] proposed an optimization scheme to adjust the backoff exponent that was based on the clear channel assessment (CCA) results, which improves throughput and energy efficiency of the network. Y. Zhang et al. [14] presented a blind adaptive access parameter optimization algorithm to improve energy efficiency. B. Khandish et al. [15] adjusted the minimum value of the random backoff by using CCA results to improve network throughput. J. He et al. [16] studied the impact of the parameters of the slotted CSMA-CA mechanism on throughput by using two Markov chains. I. Bouazzi et al. [17] dynamically adjusted MAC parameters, based on the length of each sensor queue, and analyzed delay and throughput. G. Boudour et al. [18] proposed an algorithm that adjusts the competition parameters according to the observed busy channel and transmission failure rate. S. Moulik et al. [19] used a superframe duration–beacon interval ratio in an analysis model, which shows that delay can be reduced by 35 percent by adjusting some MAC parameters. Khanafer et al. [20] presented the adaptive backoff algorithm (ABA), in which the size of contention window is adaptively determined based on the collisions experienced by the nodes. Faridi et al. [21] scrutinized the assumptions made in the prevalent Markovian models and singled out the assumptions that influence performance metrics, including throughput, delay, power consumption, collision probability and packet discard probability. Azdad et al. [22] focused on understanding MAC parameters and manipulating the configuration of MAC parameters to support higher data rates in wireless body area networks (WBANs). Moulik et al. [23] presented a mechanism to ensure the reliable transmission of critical packets in WBANs that adopt the IEEE 802.15.4 standard. Alaparthi et al. [24] studied the suitability of CSMA-CA for WSN. Nabila et al. [25] enhanced the backoff strategy for the slotted CSMA-CA defined in the IEEE 802.15.4 standard in order to provide nodes with fair channel access. Singh et al. [26] proposed the “device registration” algorithm to support the applications that generate time-sensitive and sporadic data by modifying the parameters within the framework of the existing MAC. Aboubakar et al. [27] proposed an efficient approach for configuring the IEEE 802.15.4 MAC parameters, in which the predictive feature of machine learning algorithms is used to determine the IEEE 802.15.4 MAC parameters. Musaddiq et al. [28] applied a reinforcement learning (RL) mechanism to efficiently handle the channel access mechanisms. Alimorad et al. [29] presented a numerical MAC optimization algorithm for beacon-enabled IEEE 802.15.4-based WBANs to optimize delay, reliability and energy trade-offs, based on the existing traffic and QoS requirements.

In contrast to the existing schemes, our ECCE targets the maximum CCE by optimizing the three key parameters of *macMaxCsmaBackoffs*, *macMinBe* and *macMaxBe* [4] in the CSMA-CA mechanism to reduce delay and improve throughput.

## 3. Background and Problem Definition

In a slotted 802.15.4 network, each node abides by the CSMA-CA mechanism in each data transmission trial. With the CSMA-CA mechanism, a node maintains the three key variables of *NB*, *CW* and *BE*, which stand for the number of backoffs, contention window and backoff exponent, respectively. The variable *NB* holds the number of CSMA-CA algorithms being conducted to back off the current transmission. The variable *CW* defines the number of successive backoff periods in which the channel is clear before the transmission can initiate. The variable *BE* sets $2^{BE}-1$ backoff periods, from which the node randomly picks one [3].

**Figure 1:**
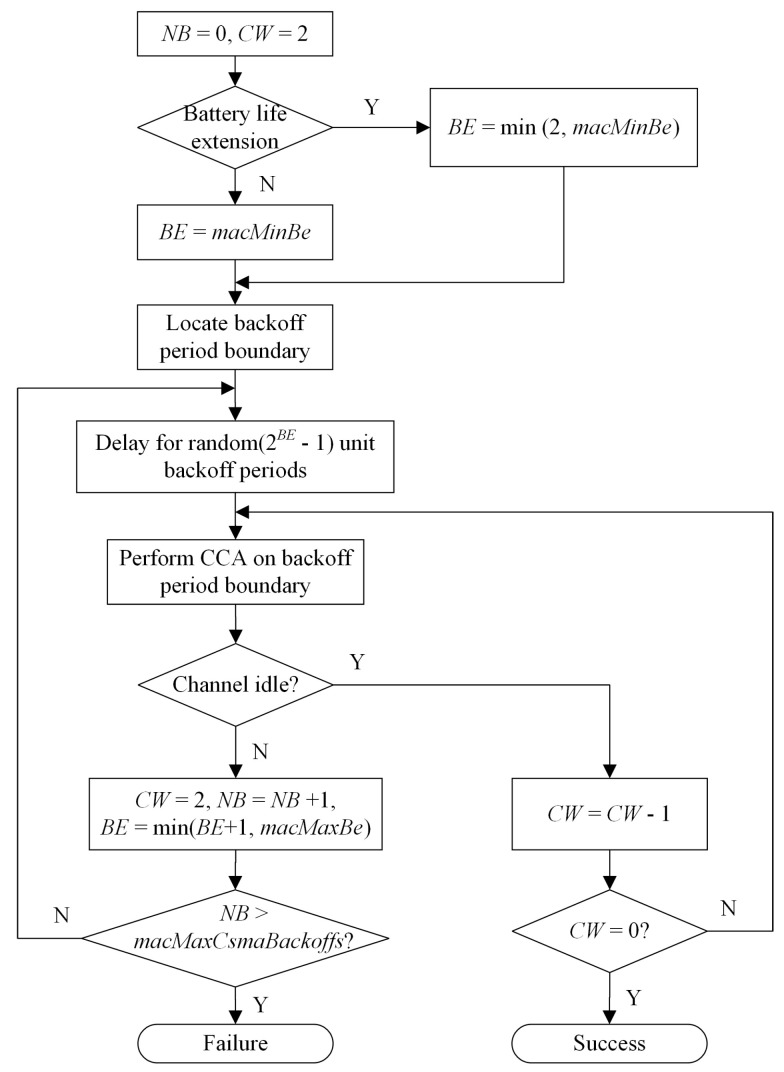
The procedure of the CSMA-CA algorithm [2,3].

The procedure of the slotted CSMA-CA channel access mechanism is shown in Figure 1. In the figure, *macMaxBe*, *macMinBe* and *macMaxCsmaBackoffs* are the three key parameters defined in the MAC layer, which represent the maximum of *BE*, the minimum of *BE* and the upper bound on the number of CSMA-CA algorithms being conducted, respectively. In the procedure, variables *NB* and *CW* are initialized to zero and two, respectively. Then, variable *BE* is set to the lesser of *macMinBe* and two, depending on battery life extension information. After locating or aligning the backoff period boundary, the node randomly picks a number, say *x*, from the set $\left\{1,2,\cdots ,2^{BE}-1\right\}$. Then, the node keeps silent until the boundary of the *x*-th and the (x+1)-th backoff periods, when the node performs a clear channel assessment (CCA) to see whether the channel is idle or not. If it is, the node checks whether the beginning of the next backoff period is still idle. Only when *CW* (i.e., two) successive backoff periods are idle can the node transmit its data. Otherwise, the node prepares the next random backoff period after resetting CW to two and increasing *NB* by one, except when *BE* reaches its limitation *macMaxBe*.

Obviously, an increase in *BE* may increase the node’s waiting time before gaining the channel because the node picks a random number from a set with nearly twice as many backoff periods as the previous one. It should be noted that *NB*, *CW* and *BE* are reset whenever the node succeeds in gaining the channel or fails after reaching the *macMaxCsmaBackoffs* number of random backoffs.

Although the ranges of the *macMinBe*, *macMaxBe* and *macMaxCsmaBackoffs* parameters are defined in the IEEE 802.15.4 standard, the nodes operating within this standard are faced with the problem of lacking the knowledge of how to set the optimal values for these parameters. This problem will be addressed in the next section.

## 4. The Proposed Scheme with Mathematical Model

### 4.1. The ECCE Scheme

When a node intends to deliver a packet, it contends for the channel via a CSMA-CA mechanism that incorporates the three key parameters of *macMaxCsmaBackoffs*, *macMinBE* and *macMaxBE*.

In the proposed ECCE scheme, when the node performs a CCA, the node calculates the probability of the CCA sensing a busy channel, denoted by *h*, according to the following procedure. The node uses ϰ to hold the total number of times that the node performs a CCA and $\varkappa_{b}$ to hold the number of times that the node has sensed a busy channel with the CCA. The probability *h* is initialized to zero. Each time the node conducts a CCA, ϰ is always increased by one and $\varkappa_{b}$ is only increased by one when the CCA senses a busy channel. Then, the node updates $h=\varkappa_{b}/\varkappa$.

In the ECCE scheme, when a node fails in gaining the channel, it replaces the three key parameters in the CSMA-CA with the solution of the optimization problem in (32) in Section 4.4, which aims to maximize the CCE as defined in Section 4.3.

### 4.2. Markov Chain for the CSMA-CA Mechanism

In 802.15.4 wireless networks, a node transmits packets one after another. It can be seen from the CSMA-CA mechanism in Figure 1 that, for a given packet to be delivered, the node attempts to contend for the channel at most *K* times under the CSMA-CA mechanism, where K= *macMaxCsmaBackoffs*. With the CSMA-CA mechanism, we define the node’s state as follows:

- State “A(i,j)” represents that the node intends to gain the channel on the *i*-th attempt (i.e., it fails in the previous i−1 attempts) and it performs a CCA for the first time at the beginning of the *j*-th backoff period, where i∈{1,2,⋯,K}, $j\in \{1,2,\cdots ,n_{i}\}$. Here, from Figure 1, we have:
  (1) $$\begin{array}{cc} n_{i} & =\min\{2^{X_{0}+i-1}-1,\;2^{X_{1}}-1\}\\ \; & \;\;\;(i=1,2,\cdots ,K), \end{array}$$
  where $X_{0}=\mathrm{macMinBe}$ and $X_{1}=\mathrm{macMaxBe}$. From the IEEE 802.15.4 standard [3], $X_{1}\in \left\{3,4,\cdots ,8\right\}$ is defaulted to five, $X_{0}\in \left\{1,2,\cdots ,X_{1}\right\}$ is defaulted to three and K∈{1,2,3,4,5} is defaulted to four. In addition, we introduce:
  (2) $$s_{\;K}=\sum_{i=1}^{K}n_{i}.$$
- State “B(i,j)” represents that the node intends to gain the channel on the *i*-th attempt and it performs a CCA for *the second time* at the beginning of the (*j* + 1)-th backoff period (this is the equivalent of performing a CCA at the end of *j*-th backoff period because the CCA is conducted at the boundary of two neighboring backoff periods), where i∈{1,2,⋯,K}, $j\in \{1,2,\cdots ,n_{i}\}$;
- State “$C_{0}$” represents that the node has prepared channel contention for transmitting the packet;
- State “$C_{i}\left(i\geq 1\right)$” represents that the channel is not idle according to the CCA on the *i*-th attempt;
- State “*S*” represents that the node has succeeded in gaining the channel;
- State “*H*” represents that the node has halted channel contention as the number of attempts reaches its maximum.

**Figure 2:**
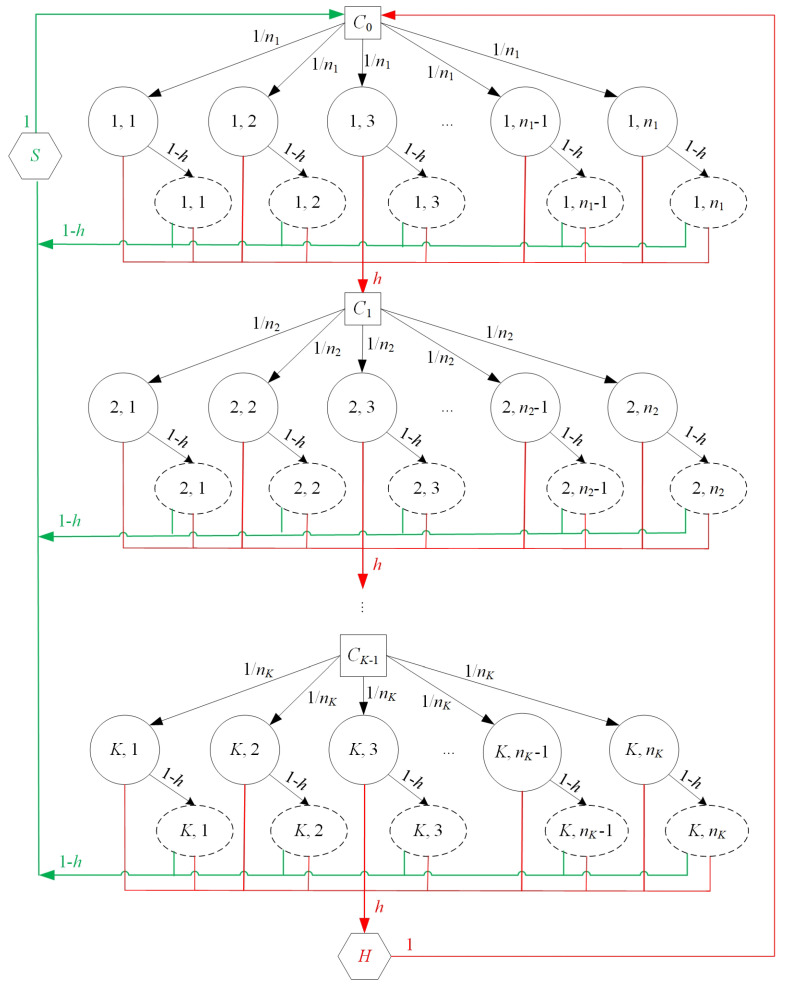
The state transition diagram.

The transitions of the above-defined states are shown in Figure 2, where a circle with the integers *i* and *j* inside stands for state A(i,j), a dotted ellipse with the integers *i* and *j* stands for state B(i,j), a square with $C_{i}$ inside stands for state $C_{i}$, a hexagon with the letter “*S*” stands for state *S* and a hexagon with the letter “*H*” stands for state *H*. From the state transition diagram in Figure 2, we calculated the state transition probabilities as follows:

(3) $$\begin{array}{cc} \; & Pr\left\{A_{i,j}|C_{i-1}\right\}=\frac{1}{n_{i}},\\ \; & \;\;\;i=1,2,\cdots ,K,\;j=1,2,\cdots ,n_{i}; \end{array}$$

(4) $$\begin{array}{cc} \; & Pr\{B_{i,j}|A_{i,j}\}=1-h,\\ \; & \;\;\;i=1,2,\cdots ,K,\;j=1,2,\cdots ,n_{i}; \end{array}$$

(5) $$\begin{array}{cc} \; & Pr\{C_{i}|A_{i,j}\}=h,\\ \; & \;\;\;i=1,2,\cdots ,K-1,\;j=1,2,\cdots ,n_{i}; \end{array}$$

(6) $$\begin{array}{cc} \; & Pr\left\{H|A_{K,j}\right\}=h,\;j=1,2,\cdots ,n_{K}; \end{array}$$

(7) $$\begin{array}{cc} \; & Pr\{C_{i}|B_{i,j}\}=h,\\ \; & \;\;\;i=1,2,\cdots ,K-1,\;j=1,2,\cdots ,n_{i}; \end{array}$$

(8) $$\begin{array}{cc} \; & Pr\left\{H|B_{K,j}\right\}=h,\;j=1,2,\cdots ,n_{K}; \end{array}$$

(9) $$\begin{array}{cc} \; & Pr\{S|B_{i,j}\}=1-h,\\ \; & \;\;\;i=1,2,\cdots ,K,\;j=1,2,\cdots ,n_{i}; \end{array}$$

(10) $$\begin{array}{cc} \; & Pr\{C_{0}|H\}=1; \end{array}$$

(11) $$\begin{array}{cc} \; & Pr\{C_{0}|S\}=1. \end{array}$$

As mentioned previously, *h* is the probability of the CCA sensing that the channel is busy when the node tries to gain the channel.

The state transition diagram is explained as follows. When the node intends to transmit a packet, then the node stays in state $C_{0}$ as it needs to contend for the channel and thus, it experiences a random backoff period in which there are $n_{1}$ integers ($1,2,\cdots ,n_{1}$) with each being equally likely to be picked according to the CSMA-CA mechanism. Therefore, it has the probability of $1/n_{i}$ that the node transfers from state $C_{0}$ to state A(i,j). Equivalently, Equation (3) holds. In the case of the node being in state A(1,j), if the CCA indicates that the channel is idle, the node needs to perform the CCA again at the beginning of the next backoff period due to CW=2, i.e., its state changes to B(i,j). Otherwise, its state becomes $C_{1}$, which indicates that the node fails in gaining the channel on the first attempt. That is, $Pr\{B_{1,j}|A_{1,j}\}=1-h$ and $Pr\{C_{1}|A_{1,j}\}=h$ ($j=1,2,\cdots ,n_{1}$). Similarly, we can analyze the case when the node is in state A(i,j)(i>1), which leads to Equations (4) and (5). In the case of the node being in state A(K,j), if the channel is sensed to be busy, which happens with probability of *h*, it causes the node to be in state *H* (i.e., the node senses the channel is in use on the *K*-th attempt and thus, the node halts channel contention). This yields (6). In the case of the node being in state B(i,j), if the channel is sensed to be busy, which occurs with the probability of *h*, it brings the node to either state $C_{i}$ if i<K or state *H* if i=K, whereas a clear channel brings the node to the success state *S*. Thus, Equations (7)–(9) hold true. In the case of the node being in states *S* or *H*, which indicate the completion of the channel contention for the current packet, the node’s state changes back to $C_{0}$, i.e., the node prepares to transmit the next packet in its transmission buffer. This is reflected in (10) and (11).

$A_{i,j}$, $B_{i,j}$, $C_{i}$, *H* and *S* denote the probabilities of the node being in states $a_{i,j}$, $b_{i,j}$, $c_{i}$, $p_{h}$ and $p_{s}$, respectively. From Figure 2, after considering all states, we used the following equations for the Markov chain, according to the flow balance:

(12) $$\begin{array}{cc} \; & c_{0}=p_{s}+p_{h}; \end{array}$$

(13) $$\begin{array}{cc} \; & a_{i,j}=c_{i-1}\;\frac{1}{n_{i}},\\ \; & \;i=1,2,\cdots ,K,j=1,2,\cdots ,n_{i}; \end{array}$$

(14) $$\begin{array}{cc} \; & b_{i,j}=\left(1-h\right)a_{i,j},\\ \; & \;i=1,2,\cdots ,K,j=1,2,\cdots ,n_{i}; \end{array}$$

(15) $$\begin{array}{cc} \; & c_{i}=h\left(\sum_{j=1}^{n_{i}}a_{i,j}+\sum_{j=1}^{n_{i}}b_{i,j}\right),\\ \; & \;i=1,2,\cdots ,K-1; \end{array}$$

(16) $$\begin{array}{cc} \; & p_{h}=h\left(\sum_{j=1}^{n_{\;K}}a_{\;K,j}+\sum_{j=1}^{n_{\;K}}b_{K,j}\right); \end{array}$$

(17) $$\begin{array}{cc} \; & p_{s}=\left(1-h\right)\sum_{i=1}^{K}\sum_{j=1}^{n_{i}}b_{i,j}. \end{array}$$

Obviously, we used the following normalization equation for all probabilities:

(18) $$\begin{array}{cc} \; & p_{s}+p_{h}+\sum_{i=0}^{K-1}c_{i}+\sum_{i=1}^{K}\sum_{j=1}^{n_{i}}a_{i,j}+\sum_{i=1}^{K}\sum_{j=1}^{n_{i}}b_{i,j}=1. \end{array}$$

From Equations (13)–(18), we calculated:

(19) $$\begin{array}{c} A\;\vec{P}=\vec{e}, \end{array}$$

where $\vec{P}$ and $\vec{e}$ are vectors, with each having $K+2(s_{\;K}+1)$ elements, and A is a square matrix with each column having the same dimension as vector $\vec{P}$. Specifically, vector $\vec{e}$ contains elements of 0 s, except for the last element of one, i.e., $\vec{e}={\left(0,0,\cdots ,0,1\right)}^{T}$. In addition, vector $\vec{P}$ consists of all state probabilities as $\vec{P}={\left(p_{s},p_{h},\tilde{c},{\tilde{a}}_{1},{\tilde{a}}_{2},\cdots ,{\tilde{a}}_{K},{\tilde{b}}_{1},{\tilde{b}}_{2},\cdots ,{\tilde{b}}_{K}\right)}^{T}$, in which we define row vectors $\tilde{c}=\left(c_{0},c_{1},\cdots ,c_{K\;-1}\right)$, ${\tilde{a}}_{i}=\left(a_{i,1},a_{i,2},\cdots ,a_{i,n_{i}}\right)$ and ${\tilde{b}}_{i}=\left(b_{i,1},b_{i,2},\cdots ,b_{i,n_{i}}\right)$, where i=1,2,⋯,K. Moreover:

(20) $$\begin{array}{c} A=\left[\begin{array}{cccc} \; & B_{s_{\;K}\times K} & -I_{s_{\;K}} & \\ \; & \; & \left(1-h\right)I_{s_{\;K}} & -I_{s_{\;K}}\\ \; & F_{(K-1)\times K} & G_{\left(K-1\right)\times s_{\;K}} & G_{\left(K-1\right)\times s_{\;K}}\\ -I_{2} & \; & D_{2\times s_{\;K}} & E_{2\times s_{\;K}}\\ {\tilde{1}}_{2} & {\tilde{1}}_{K} & {\tilde{1}}_{s_{\;K}} & {\tilde{1}}_{s_{\;K}} \end{array}\right]. \end{array}$$

In (20), the last row consists of all 1 s, which corresponds to (18). Here, we define ${\tilde{1}}_{x}$ as the row vector with *x* 1 s. In addition, we define ${\tilde{0}}_{x}$ as the row vector with *x* 0 s. Meanwhile, we define ${\vec{0}}_{x}$ and ${\vec{1}}_{x}$ as the *x*-element column vectors consisting of 0 s and 1 s, respectively. The matrix blocks in (20) are introduced as follows. $I_{x}$ stands for the *x*-th order identity matrix (its diagonal elements are 1 s and the other elements are 0 s). Additionally, we calculated:

(21) $$\begin{array}{c} B_{s_{\;K}\times K}=\left[\begin{array}{cccc} \frac{{\vec{1}}_{n_{1}}}{n_{1}} & 0 & \cdots & 0\\ 0 & \frac{{\vec{1}}_{n_{2}}}{n_{2}} & \cdots & 0\\ \vdots & \vdots & \ddots & \vdots \\ 0 & 0 & \cdots & \frac{{\vec{1}}_{n_{K}}}{n_{K}} \end{array}\right], \end{array}$$

(22) $$\begin{array}{c} D_{2\times s_{\;K}}=\left[\begin{array}{cc} {\tilde{0}}_{s_{\;K}-n_{\;K}} & {\tilde{0}}_{n_{\;K}}\\ {\tilde{0}}_{s_{\;K}-n_{\;K}} & h\;{\tilde{1}}_{n_{\;K}} \end{array}\right], \end{array}$$

(23) $$\begin{array}{c} E_{2\times s_{\;K}}\;=\;\left[\begin{array}{cc} \left(1-h\right){\tilde{1}}_{s_{\;K}-n_{\;K}} & \left(1\;-\;h\right){\tilde{1}}_{n_{\;K}}\\ {\tilde{0}}_{s_{\;K}-n_{\;K}} & h{\tilde{1}}_{n_{\;K}} \end{array}\right], \end{array}$$

(24) $$\begin{array}{c} F_{(K-1)\times K}=\left[\begin{array}{cc} {\vec{0}}_{K-1} & -I_{K-1} \end{array}\right], \end{array}$$

(25) $$\begin{array}{c} G_{\left(\;K\;-\;1\right)\times s_{\;K}}=\left[\begin{array}{ccccc} h{\tilde{1}}_{n_{1}} & {\tilde{0}}_{n_{2}} & \cdots & {\tilde{0}}_{n_{\;K-\;1}} & {\tilde{0}}_{n_{\;K}}\\ {\tilde{0}}_{n_{1}} & h{\tilde{1}}_{n_{2}} & \cdots & {\tilde{0}}_{n_{\;K-\;1}} & {\tilde{0}}_{n_{\;K}}\\ \vdots & \ddots & \vdots & \vdots & \vdots \\ {\tilde{0}}_{n_{1}} & {\tilde{0}}_{n_{2}} & \cdots & h{\tilde{1}}_{n_{\;K-\;1}} & {\tilde{0}}_{n_{\;K}} \end{array}\right]. \end{array}$$

From Equation (19), we obtained $\vec{P}=A^{-1}\vec{e}$, which contains all state probabilities.

With the derived probabilities $b_{i,j}$s, from (17), we obtained the probability of successfully gaining the channel as follows:

(26) $$\begin{array}{c} p_{s}\left(K,X_{0},X_{1}\right)=\left(1-h\right)\sum_{i=1}^{K}\sum_{j=1}^{n_{i}}b_{i,j}, \end{array}$$

where the success probability is expressed as function of *K* and $n_{i}$ is given in (1).

### 4.3. Statistics for the CSMA-CA Mechanism

Using the probabilities derived in the previous section, we could obtain some statistics under the CSMA-CA mechanism.

Firstly, the expected number of times that the node fails in gaining the channel for transmitting a packet was:

(27) $$\begin{array}{c} N_{f}\left(K,X_{0},X_{1}\right)=\sum_{i=1}^{K-1}i\left(\frac{c_{i}}{\sum_{j=1}^{K-1}c_{j}}\right)=\frac{\sum_{i=1}^{K-1}ic_{i}}{\sum_{j=1}^{K-1}c_{j}}, \end{array}$$

where $c_{i}/\sum_{j=1}^{K-1}c_{j}$ is the probability that the node senses a busy channel on the *i*-th trial, which is a function of $X_{0}$ and $X_{1}$.

Secondly, in the CSMA-CA algorithm, the node needs to conduct the CCA twice to check whether the channel is idle. On the *i*-th attempt to gain the channel, there are $n_{i}$ backoff periods from which the node picks one with an identical probability of $1/n_{i}$. Thus, the node averagely suffers from $\left(1+2+\cdots +n_{i}\right)/n_{i}=\left(n_{i}+1\right)/2$ backoff periods before the first CCA. After the backoff, the node performs the first CCA. In the case of the first CCA sensing that the channel is idle, which happens with probability of 1−h, the second CCA is conducted, which consumes one more backoff period regardless of whether the second CCA senses the idle channel or not. Hence, the double CCA consumes a time of (1−h)×1+h×0=1−h backoff period, on average. As a result, on the *i*-th attempt, the expected number of backoff periods that the node experiences is $\left(n_{i}+1\right)/2+1-h=\left(n_{i}-2h+3\right)/2,\;i=1,2,\cdots ,K$.

By noticing that the node attempts to gain the channel only in states $C_{0},C_{1},\cdots ,C_{\;K\;-\;1}$, we obtained the probability of the node conducting the *i*-th attempt as $c_{i-1}/\sum_{j=0}^{K-1}c_{j},\;i=1,2,\cdots ,K$.

Thus, the expected number of backoff periods that the node experiences for transmitting a packet was:

(28) $$\begin{array}{cc} N_{bp}\left(K,X_{0},X_{1}\right) & =\sum_{i=1}^{K}\frac{c_{i-1}}{\sum_{j=0}^{K-1}c_{j}}\left(\frac{n_{i}-2h+3}{2}\right)\\ \; & =\frac{\sum_{i=1}^{K}c_{i-1}\left(n_{i}-2h+3\right)}{2\sum_{j=0}^{K-1}c_{j}}. \end{array}$$

In addition, the expected number of backoffs that the node experiences for transmitting a packet, which is equal to the expected number of transmission trials, was:

(29) $$\begin{array}{c} N_{b}\left(K,X_{0},X_{1}\right)=\sum_{i=1}^{K}i\frac{c_{i-1}}{\sum_{j=0}^{K-1}c_{j}}=\frac{\sum_{i=1}^{K}ic_{i-1}}{\sum_{j=0}^{K-1}c_{j}}. \end{array}$$

Thirdly, in order to transmit a packet, the node can use the channel once if it succeeds in gaining the channel, which occurs with probability $p_{s}\left(\cdot \right)$, and it does not use the channel otherwise. Hence, the expected number of times that the node gains the channel successfully is $p_{s}\left(\cdot \right)\times 1+\left[1-p_{s}\left(\cdot \right)\right]\times 0=p_{s}\left(\cdot \right)$. By considering the expected time consumed by the node in contending for the channel as $N_{bp}\left(\cdot \right)\sigma$, where σ is the duration of one backoff period set to *aUnitBackoffPeriod*, a parameter defined in the MAC layer of the IEEE 802.15.4 standard, we defined *Channel Contention Efficiency (CCE)* as:

(30) $$\begin{array}{c} \delta \left(K,X_{0},X_{1}\right)=\frac{p_{s}|\left(K,X_{0},X_{1}\right)}{N_{bp}\left(K,X_{0},X_{1}\right)\sigma }. \end{array}$$

The CCE in (30) can be explained as the number of times that the node successfully gained the channel per unit of backoff time.

Using (26) and (28), we calculated the CCE to be as follows:

(31) $$\begin{array}{c} \delta \left(K,X_{0},X_{1}\right)=\frac{2\left(1-h\right)\sum_{j=0}^{K-1}c_{j}\sum_{i=1}^{K}\sum_{j=1}^{n_{i}}b_{i,j}}{\sigma \sum_{i=1}^{K}c_{i-1}\left(n_{i}-2h+3\right)}. \end{array}$$

### 4.4. Optimization Problem

Our scheme aims to maximize the CCE in (31). We formulated the optimization problem (OP) as follows:

(32) $$\begin{array}{cc} \; & \max\;\delta (K,X_{0},X_{1})\\ \; & w.r.t.:K,X_{0},X_{1}\\ \; & s.t.\left\{\begin{array}{c} K\in \{1,2,3,4,5\};\\ X_{1}\in \left\{3,4,\cdots ,8\right\};\\ X_{0}\in \left\{1,2,\cdots ,X_{1}\right\}. \end{array}\right. \end{array}$$

The constraints in the above optimization problem are from IEEE 802.15.4 standard [3].

Considering that the number of triple $\left(K,X_{0},X_{1}\right)$s that satisfy the constraints is quite small, we used an exhaustive search to find the solution to the optimization problem.

## 5. Performance Evaluation

In this section, we present the simulation results to evaluate the effectiveness of the proposed ECCE. The simulation programs were written in MATLAB.

We assumed that there are *m* nodes in the 802.15.4 WSN. Due to the focus of this paper being on channel contention through allowing the nodes to set the three key parameters of *macMinBe*, *macMaxBe* and *macMaxCsmaBackoffs* for the CSMA-CA mechanism, we did not consider the topology of the WSN. Instead, each of the *m* nodes had a random traffic, i.e., the arrival time of a data packet was a random variable; and a packet arrival triggered the node to contend for the channel. We let each node apply the ECCE scheme to dynamically adjust its own parameters for *macMinBe*, *macMaxBe* and *macMaxCsmaBackoffs* through the optimization problem in (32).

Considering that Gamma distribution has two parameters that can be adjusted to fit most distributions, we assumed that data packet arrivals at a node follow a Gamma distribution with a shape parameter α and scale parameter β, which has the probability density function of:

(33) $$\begin{array}{c} f\left(x\right)=\left\{\begin{array}{cc} \frac{1}{\beta^{\alpha }\Gamma \left(\alpha \right)}x^{\alpha -1}e^{{}^{-\frac{x}{\beta }}}, & x\geq 0;\\ 0, & x<0. \end{array}\right. \end{array}$$

Here, $\Gamma \left(\alpha \right)=\int_{0}^{\infty }e^{-y}y^{\alpha -1}dy$. The mean and variance of the above distribution were αβ and $\alpha \beta^{2}$, respectively. Additionally, MATLAB adopted the probability density function in (33).

In addition, we used Gam(α,β) to represent the function that produced a random number, obeying the Gamma distribution with parameters of α and β.

We let σ be the duration of backoff period, i.e., σ=aUnitBackoffPeriod, which is a parameter defined in the MAC layer of the IEEE 802.15.4 standard.

**Figure 3:**
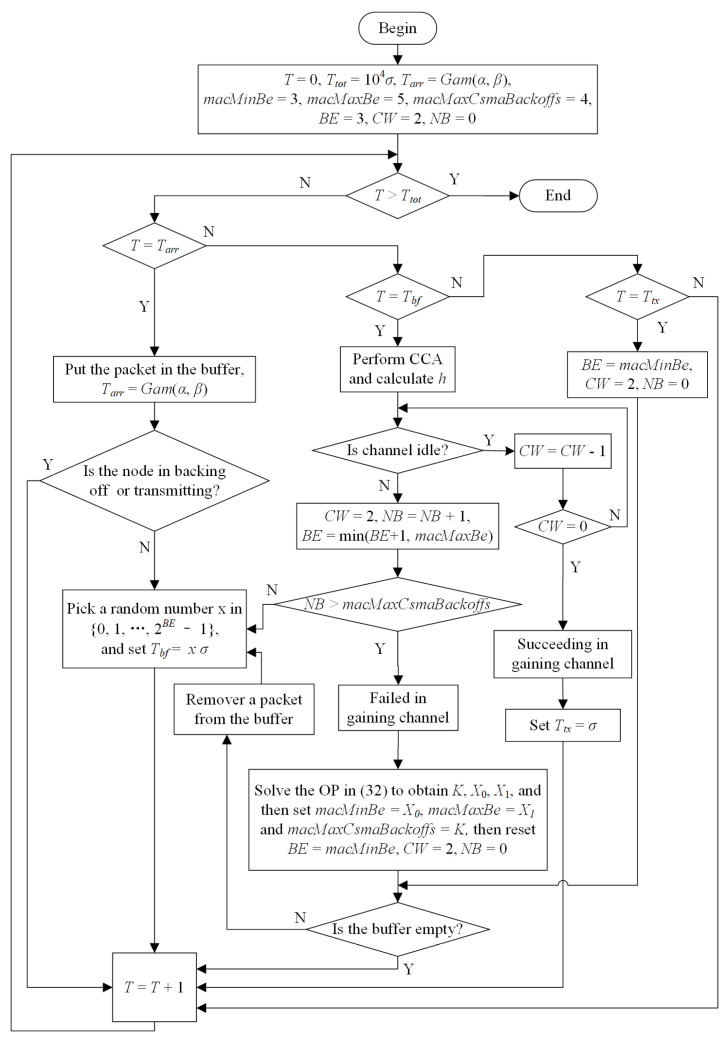
The procedure of the ECCE simulation logic.

The process of simulation proceeded microsecond by microsecond. The logic of mimicking the ECCE is shown in Figure 3. The notations in the figure are defined as follows: *T* and $T_{tot}$ stand for current time and the total simulation time, respectively; and $T_{arr}$, $T_{bf}$ and $T_{tx}$ represent the packet arrival time, backoff end time and transmission end time, respectively.

The simulation logic can be outlined as follows:

(1) Initialize the simulation parameters and variables;
(2) Advance the time on the basis of microseconds. At each microsecond, first check whether a packet has arrived. If yes, keep the packet in the buffer and then consider contending for the channel for transmission in the case of the buffer being empty. Then, check whether the node is in backoff or not. In the case when a backoff ends, the node abides by the traditional CSMA-CA mechanism, starting from the CCA. Lastly, check whether the node is transmitting a packet. The completion of the transmission resets the relative parameters.

In the simulation process, our optimization problem in (32) was applied when the node fails in gaining the channel so that the MAC parameters in the CSMA-CA were adjusted to adapt to channel competition.

We compared the ECCE to the CSMA-CA scheme for the 802.15.4 standard and the ABA proposed in [20] in terms of CCE, delay, percentage of reduced delay (PoRD), throughput, percentage of increased throughput (PoIT) and collision probability. Here, the PoRD is defined as the ratio $(a_{1}-a)/a$, where *a* stands for the delay of ECCE and $a_{1}$ is the delay of the CSMA-CA or the ABA; and PoIT is $\left(b-b_{1}\right)/b_{1}$, where *b* represents the throughput of ECCE and $b_{1}$ is the throughput of the CSMA-CA or the ABA. The following results are from a simulation with $10^{4}$ time slots, with each having length of 1160μs.

**Figure 4:**
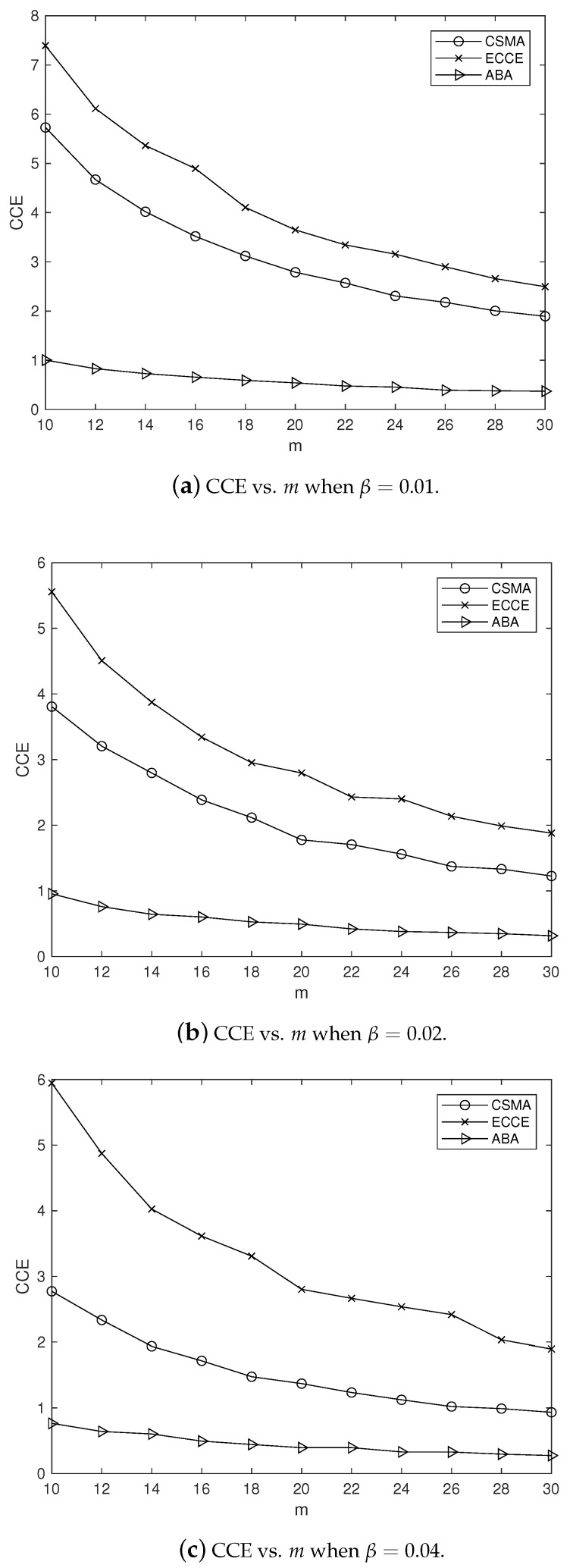
The impact of *m* on CCE when α=1.

**Figure 5:**
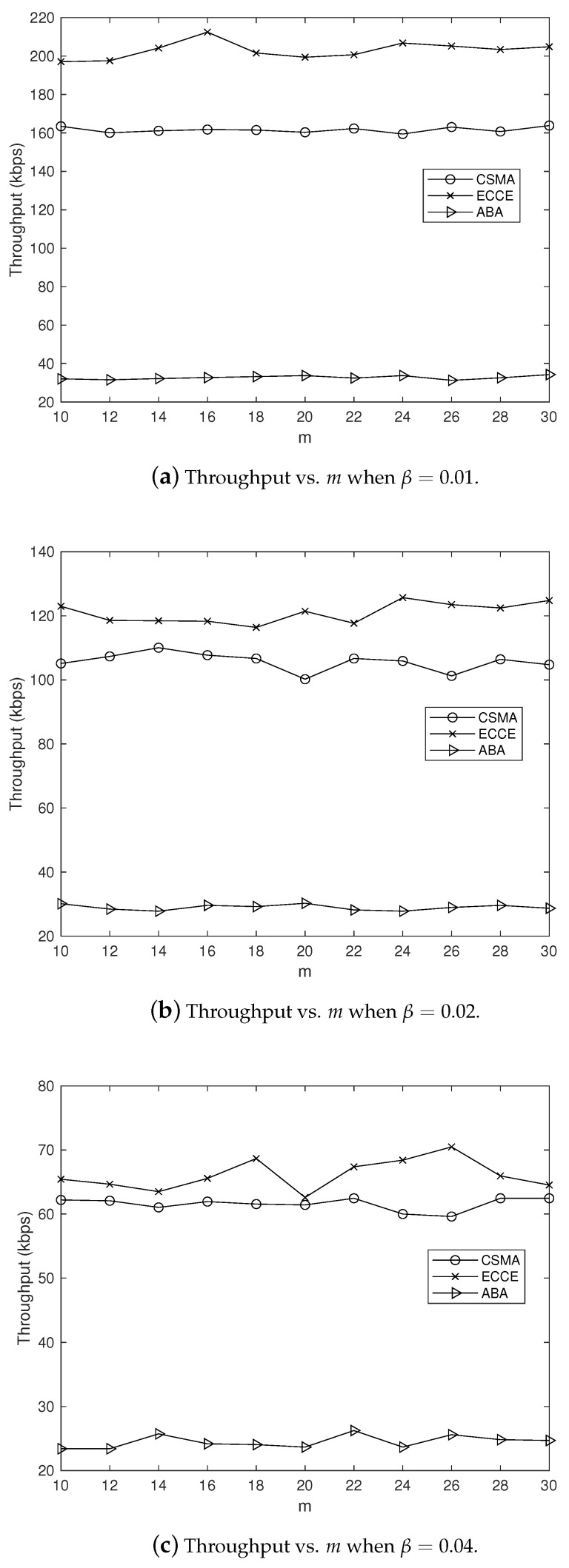
The impact of *m* on throughput when α=1.

**Figure 6:**
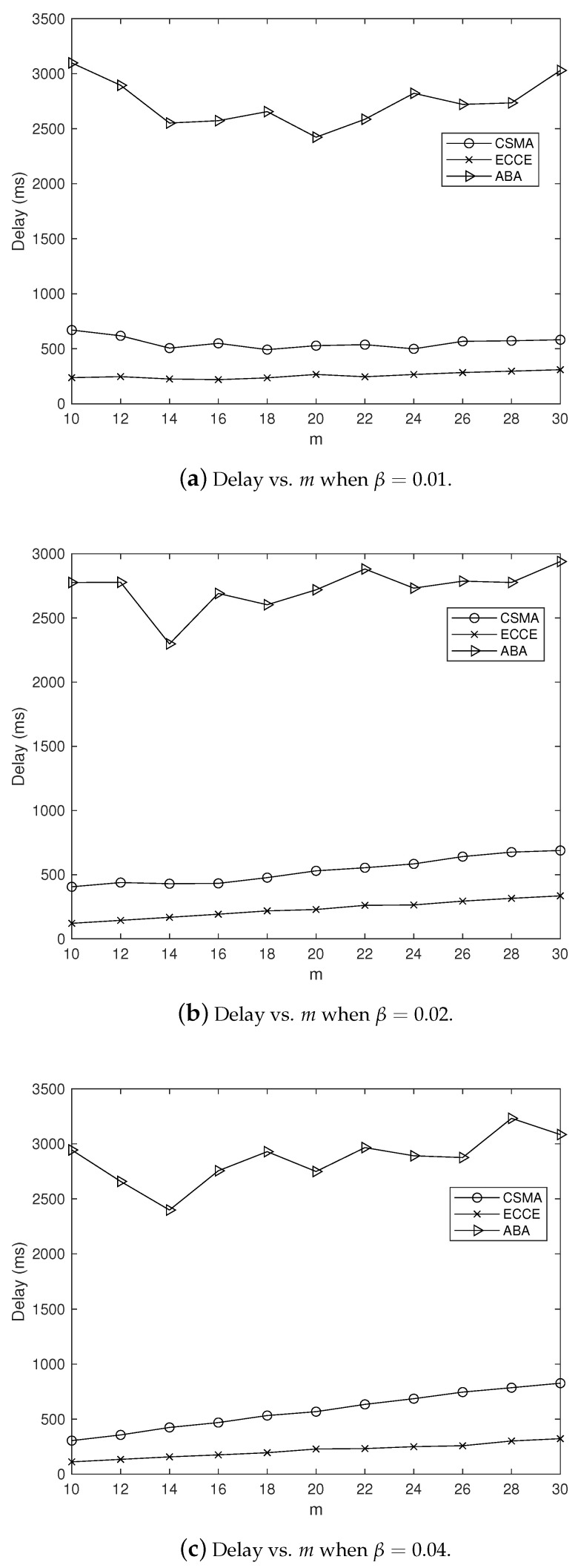
The impact of *m* on delay when α=1.

**Figure 7:**
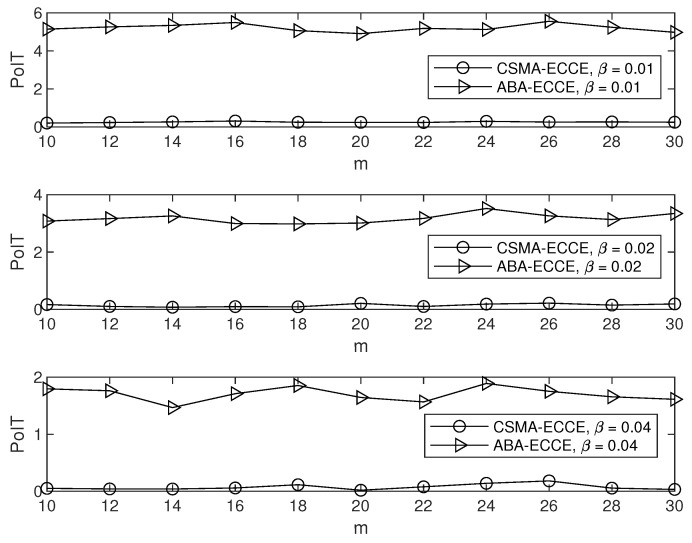
PoIT vs. *m*.

**Figure 8:**
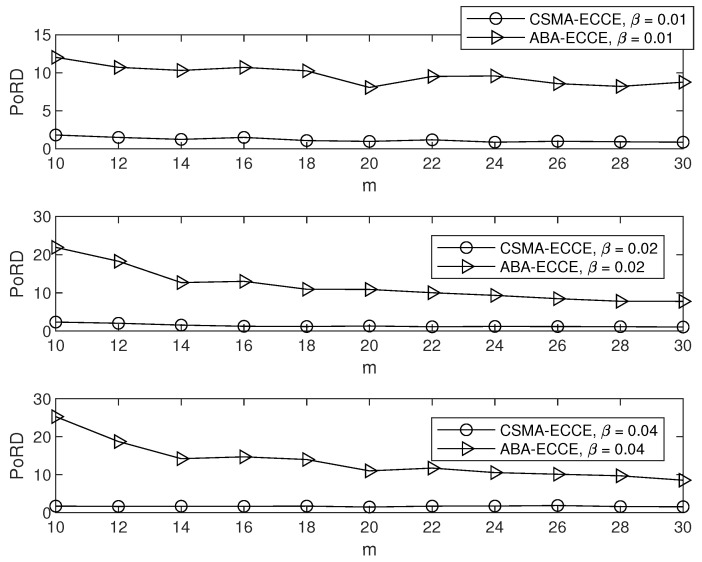
PoRD vs. *m*.

In order to clearly observe the CCE in our ECCE, we set m=10,12,⋯,30 and β=0.01,0.02,0.04 (in seconds), and fixed α=1, which yielded the simulation results in Figure 4, where the CCE is the average over the number of nodes. It can be clearly seen from Figure 4 that the ECCE had a higher CCE than that of the CSMA-CA and the ABA. This agrees with the aim of our ECCE to maximize the CCE. The same settings for the parameters m,α and β led to the delay, throughput, PoIT and PoRD shown in Figure 5, Figure 6, Figure 7 and Figure 8. From Figure 5, we can observe the following: (1) the proposed ECCE scheme outperformed the CSMA-CA and the ABA in throughput; and (2) the throughput in the ECCE nearly kept steady with the growth of the number of nodes (i.e., *m*). In Figure 6, we can observe that the ECCE scheme also outperformed the CSMA-CA and the ABA in delay and that the packet delay under the ECCE scheme gradually increased with the number of nodes, due to the fact that channel contention becomes more serious when more nodes join in contending for the channel. The reason that the proposed ECCE scheme had better throughput and delay than the other two schemes is that the ECCE scheme could adapt to the contention situation by setting the three key parameters in the CSMA-CA scheme to their respective optimal values via the optimization problem in (32). It can be seen from Figure 7 and Figure 8 that PoIT and PoRD differed with different values of parameter β. The average interval of packet arrivals obeying the probability density function in (33) was αβ. As a result, in the case of α being fixed (we set α=1), growth in β led to fewer packet arrivals in the given time period and thus, throughput decreased. Figure 7 indicates that, for a given *m*, as β grew, the PoIT comparing the ECCE to the ABA clearly reduced whereas the PoIT comparing the ECCE to the CSMA-CA varied slightly. Figure 8 shows that variation in β, i.e., traffic variation, did not affect the PoRD too much. This is because the delay of an incoming packet does not occur until the packet arrives at a node.

**Figure 9:**
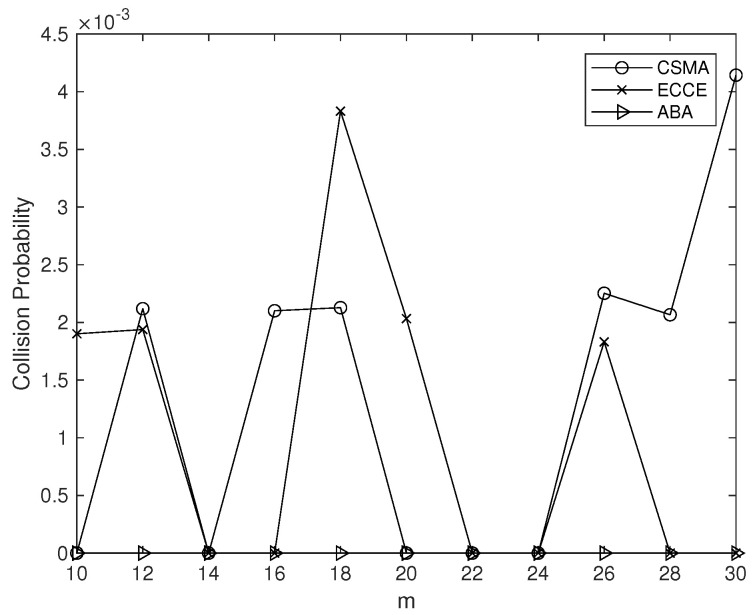
Collision probability vs. *m*.

The simulation using the same settings for the parameters showed that the collision probabilities in the ECCE, CSMA-CA and ABA were close. We present the results of the simulation when m=10,12,⋯,30 α=1 and β=0.04 in Figure 9.

In summary, the proposed ECCE could improve throughput and reduce delay by optimizing the three key parameters of the CSMA-CA scheme introduced in the MAC layer of the IEEE 802.15.4 standard.

## 6. Conclusions

Since the first publication of the IEEE 802.15.4 standard, the standard has been frequently renewed. The CSMA-CA mechanism for the node to contend for the channel, however, has remained almost unchanged. Channel contention efficiency is heavily affected by the three parameters defined in the CSMA-CA mechanism. The proposed ECCE enables a node in an 802.15.4 network to set its optimal values by solving the formulated optimization problem. With the proposed ECCE scheme, the nodes operating with the IEEE 802.15.4 standard can improve throughput and reduce packet delay. In the future, we will consider formulating an analysis model for 802.15.4 networks and setting optimal values for 802.15.4 networks in which a node has different kinds of traffic.
